# Transient Receptor Potential (TRP) Channels in Haematological Malignancies: An Update

**DOI:** 10.3390/biom11050765

**Published:** 2021-05-20

**Authors:** Federica Maggi, Maria Beatrice Morelli, Massimo Nabissi, Oliviero Marinelli, Laura Zeppa, Cristina Aguzzi, Giorgio Santoni, Consuelo Amantini

**Affiliations:** 1 Department of Molecular Medicine, Sapienza University, 00185 Rome, Italy; federica.maggi@uniroma1.it; 2 Immunopathology Laboratory, School of Pharmacy, University of Camerino, 62032 Camerino, Italy; mariabeatrice.morelli@unicam.it (M.B.M.); massimo.nabissi@unicam.it (M.N.); oliviero.marinelli@unicam.it (O.M.); laura.zeppa@unicam.it (L.Z.); cristina.aguzzi@unicam.it (C.A.); giorgio.santoni@unicam.it (G.S.); 3 Immunopathology Laboratory, School of Biosciences and Veterinary Medicine, University of Camerino, 62032 Camerino, Italy

**Keywords:** TRP, leukaemia, lymphoma

## Abstract

Transient receptor potential (TRP) channels are improving their importance in different cancers, becoming suitable as promising candidates for precision medicine. Their important contribution in calcium trafficking inside and outside cells is coming to light from many papers published so far. Encouraging results on the correlation between TRP and overall survival (OS) and progression-free survival (PFS) in cancer patients are available, and there are as many promising data from in vitro studies. For what concerns haematological malignancy, the role of TRPs is still not elucidated, and data regarding TRP channel expression have demonstrated great variability throughout blood cancer so far. Thus, the aim of this review is to highlight the most recent findings on TRP channels in leukaemia and lymphoma, demonstrating their important contribution in the perspective of personalised therapies.

## 1. Introduction

**Figure 1:**
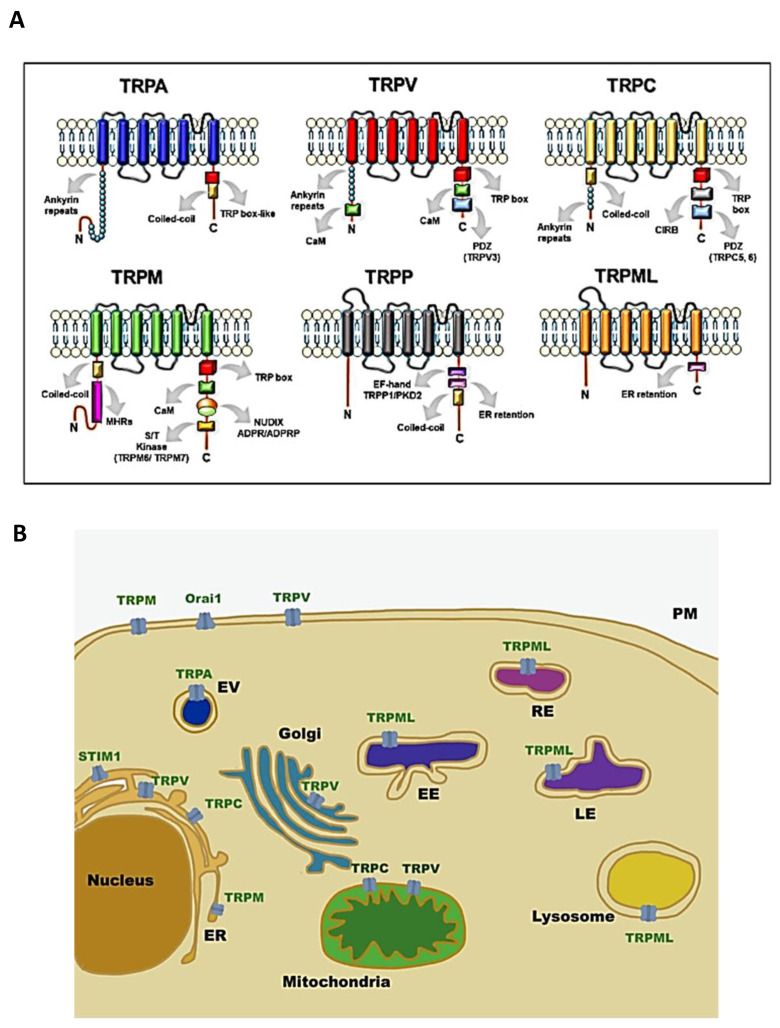
Structure and localisation of TRP channels: (**A**) structural domains and motifs in the N- and C- terminus of TRP channel subfamilies [7]; (**B**) intracellular localisation of TRP channel subfamilies and SOCE machinery. Abbreviations: TRPA, transient receptor potential ankyrin; TRPC, transient receptor potential canonical; TRPM, transient receptor potential melastatin; TRPML, transient receptor potential mucolipidin; TRPP, transient receptor potential polycystic; TRPV, transient receptor potential vanilloid; ER, endoplasmatic reticulum; EV, vesicle of exocytosis; EE, early endosome; RE, recycling endosome; LE, late endosome; PM, plasma membrane; STIM1, stromal interaction molecule 1; Orai1, calcium release-activated calcium channel protein 1.

Transient receptor potential (TRP) channels are a family of ion channels, belonging to voltage-gated superfamilies, with different physiological functions [1,2,3]. TRP channels are expressed in many organisms, from worms to mammals, and grouped in six subfamilies based on their amino acid sequence homology: canonical (TRPC), vanilloid (TRPV), melastatin (TRPM), ankyrin (TRPA), polycystic (TRPP), mucolipin (TRPML) (Figure 1) [4]. Most of TRPs are selective for cations and most of them are permeable to both monovalent cations, such as sodium (Na^+^), and divalent cations, such as magnesium (Mg^2+^) or calcium (Ca^2+^) [5]. Moreover, they can be activated by a variety of environmental stimuli such as temperature (as TRPV2), mechanical forces (as TRPA1), or plant-derived compounds such as menthol for TRPM8 or capsaicin (CPS) for TRPV1 (Table 1) [1,3,6].

Initially, it was thought that TRP channels were solely expressed on plasma membrane (PM) mediating cation entry, but many studies have demonstrated that almost all mammalian TRP channels are located in the intracellular vesicular membranes (Figure 1B). It is unclear whether TRPs are expressed in these compartments as intermediate biosynthetic pathways and then shuttled to their final location, or if they have a role as signal transducers and/or membrane trafficking regulators [28].

Above all, they are considered important Ca^2+^ selective ion channels (Table 1) which have been linked to countless physiological and pathological functions [29]. 

Indeed, calcium homeostasis shows a precise and fine regulation, and it constitutes the core of all cellular signals. Thus far, many studies have shown that cytosolic Ca^2+^ oscillations play a pivotal role in the carcinogenesis process. Tumour cells are able to remodel the Ca^2+^ signalling network and its disruption contributes to malignant phenotype development. The connection between Ca^2+^ signalling and cancer is an interesting field of study, and the emergent role of ion channels such as TRPs in regulating the Ca^2+^ network makes them promising targets for cancer treatment and care [29,30,31].

Haematological malignancies represent a mixed group of neoplasms generally divided into leukaemia and lymphoma, depending on their organs of origin. Leukaemia is based on the accumulation of immature cells by mainly affecting blood-forming cells in the bone marrow, and it is classified, according to the blood lineage, in myeloid (acute and chronic) and lymphoid (acute and chronic). Lymphoma is the neoplastic transformation of B and T lymphocytes derived from primary or secondary lymphoid organs and it is classified as non-Hodgkin and Hodgkin lymphoma [32,33].

The aim of this review is to collect and analyse the latest discoveries regarding the role of TRP channels in blood cancers starting from a previous work by Morelli et al., published in 2013 [34]. Papers are selected from 2013 up to 2021 and are outlined in Table 2.

## 2. Myeloid Acute and Chronic Leukaemia

Acute myeloid leukaemia (AML) and chronic myeloid leukaemia (CML) are grouped under myelogenous leukaemia. BCR-ABL1 fusion gene products are detected in 95% of all CML cases and thus the hallmark for this type of disease [54]. Instead, only 1% of all AML cases are positive for this condition [55]. Several other translocations (AML1-ETO, DEC-CAN, PML-RARα) and/or gain-of-function mutations (FLT3-ITD, NPM1, IDH1R132H) are present and drive the malignant phenotype in AML [56].

Previous findings have demonstrated the key role of BCR-ABL1 protein in the activation process of calmodulin kinases II (CaMKII) and IV (CaMKIV), important mediators of intracellular Ca^2+^ signalling, promoting cell proliferation [57]. Interestingly, the activation of TRPV5 and TRPV6 channels is able to arrest the activity of both CaMKs in leukaemia cells [58]. 

### 2.1. TRP Channels in AML

AML is an aggressive type of cancer identified by a block in myeloid differentiation lineage, with uncontrolled proliferation of abnormal myeloid progenitors that pile up in the bone marrow and blood flow. Its outset can be different from an underlying haematological disorder or as a consequence of prior therapy; however, mostly, it develops as a de novo disorder in healthy individuals [59,60]. Until today, several markers have been identified to improve patient’s characterisation, allowing precise treatment decisions and care [59]. Despite this, the majority of patients experience relapses, and the overall survival (OS) after 5 years is about 45%, in young patients, and less than 10%, in elderly ones [60]. In this regard, the role of TRP channels in myeloid leukaemia has been evaluated both in vitro and in vivo (Table 2). What is particularly interesting is that different TRP expressions are related to different results in AML cell fate. Specifically, TRPV expression is usually linked to cell death and cell growth, TRPM expression is associated with autophagy and NF-κB modulation, and TRPA1 expression with macrophage-related cytotoxicity. 

TRPV1 receptor has gained notoriety through the years due to its relevant role in immune response and in inflammation [61]. Several findings demonstrated that TRPV1 is expressed in the cells of both innate and adaptive immune systems such as macrophages, dendritic cells, T lymphocytes, and NK cells. However, it should also be considered that its role in the immune response has not yet been well defined, and there are conflicting data in this regard [62]. The stimulation of TRPV1 via the agonist CPS in U937 and THP-1 cell lines, especially at low doses, increases the metabolic activity, possibly mediated by mitochondrial TRPV1, without activating the cell death machinery. On the other hand, higher doses of CPS are able to induce apoptosis in a TRPV1-independent way [61,63,64]. The expression of another component of the TRPV family, TRPV2, was assessed by Siveen et al. in THP-1 and U937 cell lines. TRPV2 channel is involved in cancer proliferation, tumour resistance, and cell death [65]. TRPV2 has an important role in immune cells by acting as a molecular sensor in different immunological responses such as chemotaxis [66,67], cytokines secretion, such as TNFα and IL-6 [68], infiltration of tissues [69], phagocytosis, and degranulation [66]. Its expressions are higher (about sixfold) in leukaemia cells than in peripheral blood mononuclear cells (PBMCs). Interestingly, the THP-1 and U937 cells express two different TRPV2 isoforms: the full-length glycosylated form (f-TRPV2), and the short form (s-TRPV2), which exerts an inhibitory activity blocking the translocation of the full one [45]. The THP-1 cells express a lower amount of f-TRPV2 and higher levels of s-TRPV2 with respect to U937. Unfortunately, there are inconclusive data regarding the involvement of TRPV2 in THP-1 cell death, probably due to the low expression of the f-TRPV2, which make THP-1 more resistant to cell death induced by the TRPV2 silencing. With respect to THP-1, U937 cells are more susceptible to TRPV2 inhibition. Indeed, treatments with tranilast (TL), the TRPV2 antagonist, and SKF-96365 (SKF), a TRP inhibitor, enhance the expression of s-TRPV2 form with a parallel decrease of the f-TRPV2 expression. Additionally, TRPV2 knockdown and pharmacological inhibition slow down cell growth by triggering cell cycle arrest and apoptosis in U937 cells. This result suggests that inhibition of f-TRPV2 expression determines a reduction in its oncogenic activity. Moreover, it has been demonstrated that SFK induces p38 activation, decreases the expression of Bcl-2 and ERK 1 and 2, and increases the inactivated form of the poly(ADP-ribose) polymerase (PARP) and cleaved caspase-3 [45]. 

The expression of TRPM channels in AML has been demonstrated in U937 and HL-60 cell lines: the expression and function of TRPM2 were studied in the U937 cell line, whereas TRPM7 function was evaluated in the HL-60 cell line [48,49,70]. TRPM2 channel is important for the oxidative stress response, regulating cell survival via Ca^2+^ signalling. From data publicly available, the expression of the TRPM2 channel increases with the level of differentiation during normal haematopoiesis, and its expression is higher in AML, compared to control [70]. 

**Figure 2:**
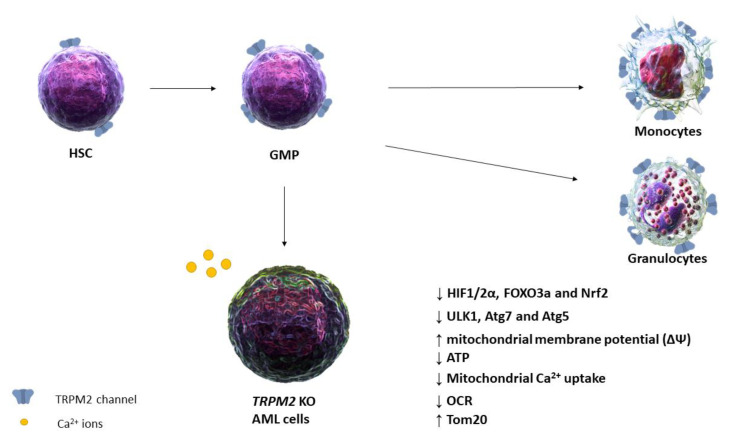
TRPM2 depletion has been correlated with the impairment of several pathways essential for the survival of leukemic cancer cells, suggesting TRPM2 as a strategic therapeutic target [48,49,70]. Abbreviations: HSC, hematopoietic stem cells; GMP, granulocyte-macrophage progenitors; AML, acute myeloid leukaemia; TRPM2, transient receptor potential melastatin 2; HIF 1/2α, hypoxia-inducible factor 1/2α; FOXO3a, forkhead box O3a; Nrf2, nuclear factor erythroid 2–related factor 2; ULK1, Unc-51 like autophagy activating kinase 1; Atg7, autophagy-related 7; Atg5, autophagy-related 5; ATP, adenosine triphosphate; OCR, oxygen consumption rate; Tom20, translocase of outer membrane 20; KO, knockout; (↑) increment; (↓) reduction.

In U937 cells, the depletion of TRPM2 substantially inhibits AML cell proliferation and promotes their sensitivity to doxorubicin through mitochondrial dysfunction, autophagy impairment, production of reactive oxygen species (ROS), and reduction of antioxidant molecules. TRPM2 is an essential ROS sensor; indeed, in *TRPM2* KO cells, mitochondrial dysfunction is pointed out by the reduction in ATP production, oxygen consumption rate (OCR), and mitochondrial Ca^2+^ uptake, with consequent increase of mitochondrial membrane potential (ΔΨ). This results in the downregulation of other proteins that take part in ROS production; in particular, hypoxia-inducible factor (HIF) 1/2α, forkhead box O3a (FOXO3a), and nuclear factor erythroid 2–related factor 2 (Nrf2) were downregulated in *TRPM2* KO U937 cells compared to the control. In addition, autophagy proteins such as Unc-51 like autophagy activating kinase 1 (ULK1), autophagy-related 7 (Atg7), and autophagy-related 5 (Atg5) were also markedly reduced, whereas the translocase of outer membrane 20 (Tom20) protein was increased in TRPM2 KO cells (Figure 2), suggesting that the depletion of TRPM2 is involved in the inhibition of autophagy [48,49]. 

Taken together, these results suggest that TRPM2 plays a leading role in AML; given that it contributes to AML survival, doxorubicin sensitivity, and myeloid cell differentiation, it can represent a promising target for innovative therapeutic strategy in AML.

Expression of TRPC channels is implicated in migration and metastatic process in numerous types of cancer [71]. Unfortunately, in HL-60 promyeloblastic cell lines, TRPC1, TRPC3, and TRPC6 receptors are barely expressed, and no data on their possible interplay with store-operated Ca^2+^ entry (SOCE) activity have been provided so far [72]. 

TRPA1 channel is widely expressed in different tissues and plays a primary role in the recognition of extracellular irritants, such as H_2_O_2_ and H_2_S [73,74]. Along with its role as a thermo- and mechanosensory channel, TRPA1 is activated by chemical stimuli and endogenous inflammatory agents such as cinnamaldehyde, allyl isothiocyanate, allicin, hydrogen peroxide, oxygen, and products of the host immune response during infection. Although TRPA1 expression in immune cells is still controversial, it has been demonstrated that TRPA1 amount on peripheral blood leukocytes correlates with pain severity and disability in rheumatoid arthritis, suggesting a role in inflammatory responses. Finally, recent data demonstrated that TRPA1 is expressed on T cells, and its endogenous activity is involved in T cell activation [75,76,77,78,79]. Tian et al. investigated the expression and function of TRPA1 in THP-1 cells. TRPA1 is expressed in THP-1-derived macrophages, both in the cytoplasm and PM, and its activation is responsible for Ca^2+^ influx, mitochondrial ROS production, mitochondrial membrane depolarisation, IL-1β release, and macrophage cytotoxicity [46,47,80,81]. 

### 2.2. TRP Channels in CML

CML is a myeloproliferative disorder first described in 1845, and it represents about 14–15% of all leukaemia cases [82]. Until now, there are no data referring to hereditary, familial, geographic, ethnic, or economic associations. The main treatments for CML include allogeneic stem cell transplantation, chemotherapy, interferon, and tyrosine kinase inhibitors (TKI) administration. CML patients show a good prognosis, but it is not uncommon to have an episode of TKI resistance or intolerance due to BCR-ABL mutations. Moreover, TKI treatments still remain not curative, despite being able to block bone marrow proliferation with effective clinical remission. Indeed, the BCR-ABL1 fusion products can remain detectable in CML patients, even after the healing [83]. For this reason, new approaches have to be improved for the control and complete removal of this disease and to avoid TKI resistance and progression to advanced stages of the disease. For these reasons, there is a need to find new drugs or at least new therapeutic targets that give the possibility of discontinuous TKI therapy.

Even in this scenario, TRP channels have been evaluated and investigated both in vitro and in vivo. Different TRP expressions lead to different phenotypes in CML; in particular, the expression of TRPV channels is linked to cell death, TRPM channels are tied to cell differentiation and proliferation, and TRPC expression is associated with cell proliferation and *BCR-ABL* gene function.

Among TRPV channels, TRPV2 has been studied in the K562 cell line. In this cell line, the expression of TRPV2 is higher, up to sixfold, than in normal PBMCs, and in THP-1 and U937 cell lines. Moreover, K562 expresses high levels of the f-TRPV2 and s-TRPV2 forms, as for THP-1 and U937 cells, but the expression of the short form is still lower with respect to the s-TRPV2 shown in the PBMCs [45,52]. TRPV2 silencing induces the loss of mitochondria membrane potential, triggering the accumulation of cells in the subG0/G1 phase, DNA fragmentation, and apoptosis activation through the mitochondrial intrinsic pathway [45]. Treatments with TRPV2 inhibitors (TL and SKF) increase the s-TRPV2 form consistently, with a concomitant decrease of the full form, which results in cell growth reduction due to apoptotic pathway activation. Indeed, active caspase-3, inactive PARP, γ-H_2_AX, and p38 are increased, whereas the Bcl-2 and ERK1/2 levels results are lower than in the control [45]. These data, together with the fact that the expression of TRPV2 in leukemic and normal blood cells has been demonstrated to be significantly different, suggest that TRPV2 can be considered a promising target for the treatment of myeloid leukaemia [45].

Regarding the TRPM subfamily, TRPM7 is abundantly expressed in the K562 cell line [52]. TRPM7 shows a unique structure recognised as “chanzyme” due to the contemporary presence of both channel and kinase-like domains. TRPM7 is expressed wildly in all cells, and numerous studies have demonstrated its implication in cell survival, growth, differentiation, and death [84]. In K562 cells, TRPM7 is expressed mainly in the PM and is essential for basal Ca^2+^ entry. The block or alteration of TRPM7 results in the reduction of cell proliferation and counters the increase of erythroid differentiation under hemin stimulation, through ERK activity [52]. Taken together, these data suggest that TRPM7 could be a promising checkpoint for K562 differentiation and for normal haematopoiesis.

Moreover, the expression of TRPC channels has been evaluated in CML cell models. In K562, the TRPC family is barely expressed [52]. In 32d cells (32d-p210), an interleukine-3 (IL-3)-dependent myeloid progenitor cell line which expresses the *BCR-ABL* gene, Cabanas et al. evaluated the expression of TRPC1 and its correlation with Ca^2+^ influx. The 32d-p210 cells showed reduced expression of TRPC1 and SOCE activity with respect to the wild-type cells (32d-WT). Given that previous studies demonstrated that different members of the TRPC family have been implicated in SOCE activity [85], this suggests a possible relationship between the SOCE activity and the TRPC1 expression. Two principal proteins are involved in SOCE activity: stromal interaction molecule 1 (STIM1) and the calcium release-activated calcium channel protein 1 (Orai1) [86]. These two proteins are essential for Ca^2+^ trafficking between the endoplasmic reticulum (ER) and the PM (Figure 1B). Their activation is subordinated by Ca^2+^ concentration in the ER; indeed, a decrease in Ca^2+^ concentration in ER prompts the translocation of STIM1 from ER to the ER/PM junction, where it binds Orai1 channels, increasing Ca^2+^ entry. In this regard, the TRPC1 translocation contributes to the STIM1/Orai1 co-localisation, participating in the SOCE activity amplification signal [87]. Interestingly, it has been demonstrated that TRPC1 KO reduces SOCE activity, proliferation, and migration in endothelial progenitor cells [88]. An opposite effect has been reported in 32d-p210 cells; in fact, even if the TRPC1 expression and SOCE activity are low, the rate of proliferation remains higher, compared to 32d-WT, suggesting a possible link between Ca^2+^-independent pathway activation and enhanced proliferation in 32d-p210 cells. The *BCR-ABL* gene seems to regulate SOCE activity tightly by also reducing the expression of TRPC1, protein kinase C (PKC) activity, and SOCEs network [51]. Therefore, TRPC1 seems a good candidate in CML treatment.

## 3. Acute Lymphoblastic (ALL) and Chronic Lymphocytic (CLL) Leukaemia

Lymphoid leukaemia is another type of blood cancer in which the cancerous change takes place in a type of marrow cell that normally proceeds to form lymphocytes. The most common type involves a specific subtype of lymphocyte, the B cell [33,89]. Within this group, it is possible to recognise the acute form (ALL) and the chronic form (CLL).

### 3.1. TRP Channels in ALL

The ALL represents the typical and the most common childhood cancer, with a low survival rate in children. In ALL, it is possible to recognise B-cell precursor lineage (B-ALL) or T-cell precursor lineage (T-ALL), and other multiple subtypes made of numerous somatic structural DNA rearrangements and sequence mutations. Everything converges in lymphocyte development: cytokine receptors, kinases and Ras cascade failure, tumour suppression, and chromatin modifications [33]. The past decade has highlighted more aspects in genetic leukemogenesis, helping to understand better clonal evolution, relapses, and the role of inherited genetic variants [90]. T-ALL shows high aggressiveness and resistance to common antileukemic drugs, which is why it is required to implement molecular targets pinpointing novel therapeutic targets and propose more efficient antileukemic drugs. A possible ‘Achille’s heel’ of ALL could be mitochondria. Mitochondria are essential promoters of oncogenic transformation, prompting cancer development, in addition to drug resistance. For these reasons, Ca^2+^ modulators and channels are gaining prominence in antileukemic drug treatments [91].

Among the TRPV family, TRPV1 is expressed in the Jurkat cell line and in patient-derived primary T-ALL cells. The activation of TRPV1 channels by resiniferatoxin (RTX), a potent analogue of CPS and a TRPV agonist, at 5 µM, arrests the cell cycle at G0/G1 phase, reduces cell proliferation, and induces apoptosis in both Jurkat cells and cell-derived T-ALL patients. In particular, after 6–12 h of treatment, a reduction of AKT, ERK, Notch-1, CDK2, and phospho-CDK2 protein levels, together with PTEN and p53 proteins upregulation in RTX-treated cells, has been reported [37]. Supporting data about the antioncogenic activity of TRPV1 were disclosed by Cetintas et al. In this study, CPS inhibits the proliferation of CCRF-CEM cells in a dose-dependent manner, inducing apoptosis via caspase-3 activation and reduction of Bcl-2 expression. Moreover, CPS treatment suppressed significantly the expression of many key cell signalling pathways, in particular Kirsten rat sarcoma (KRAS), protein kinase B (AKT), GRB2-associated binding protein 2 (GAB2), protein tyrosine phosphatase non-receptor type 11 (PTPN11), rapidly accelerated fibrosarcoma B (BRAF), inositol polyphosphate-5-phosphatase D (INPP5D), and mitogen-activated protein kinase 7 (MAPK7) proteins [92].

In the Jurkat cell line, TRPV2 reduces deformation stretch-activated current, which is important in cell migration, cell–cell interaction, and in cytokines secretion, such as TNFα and IL-6 [68], prompting the importance of these TRP channels in lymphocyte physiology [40].

Additionally, TRPV5 and TRPV6 functions were evaluated in Jurkat cells. In this model, the extracellular pH is able to affect the intracellular Ca^2+^ transport by TRPV5/6, their surface expression, and overall channel activity. Moreover, both TRPV5/6 channels participate in clathrin-mediated endocytosis and slightly overlap with the early endosome antigen 1 (EEA1) endosomal protein, suggesting a possible role in early endosomes formation [35,93]. In addition, TRPV6 is essential for Jurkat cell migration and oncogenic activity through lipid raft integrity [43]. 

TRPM2 is expressed in Jurkat cells, and it depends on Bcl-2 protein expression [94]. As proposed by Klumpp et al., TRPM2-mediated Ca^2+^ trafficking is implicated in DNA damage response and contributes to mitochondrial ROS formation. Exploiting untransfected and Bcl-2-transfected Jurkat cells (Jurkat-Bcl-2), it has been shown that the expression of the TRPM2 channel is higher in the control cells with respect to the transfected one. The antiapoptotic Bcl-2 protein, expressed in the ER or in the outer mitochondrial membrane, promotes the release of high levels of Ca^2+^ and, at the same time, decreases the mitochondrial ROS formation. TRPM2 is crucial in the regulation of the cell cycle in Jurkat cell; in fact, irradiated cells exploit TRPM2 to control the G2/M cell cycle arrest through CaMKII activation and inhibition of cdc25b and cyclin cdc2. *TRPM2* silencing or treatments cells with N-(p-amylcinnamoyl)anthranilic acid (ACA) or clotrimazole, two TRPM2 inhibitors, decreased the G2/M cell population by increasing the number of cells undergoing apoptosis, especially in irradiated Jurkat cells with respect to Jurkat-Bcl-2. Moreover, in Jurkat-Bcl-2 cells and in Jurkat cells treated with ACA or clotrimazole, the production of mitochondrial ROS species is lower with respect to the control, suggesting a connection between TRPM2-mediated Ca^2+^ uptake and mitochondrial ROS formation. Furthermore, it is suggested that stress-induced TRPM2 Ca^2+^ mediated signalling, and the mitochondrial protective function of Bcl-2 protein may cooperate in radiation therapy resistance in T-ALL [42]. 

In B-ALL patients, different members of the TRPC subfamily were evaluated. In particular, it was found that TRPC1, TRPC3, and TRPC4 channels are expressed in the PM of B-ALL cells. All of them are found to be hypermethylated and their expression downregulated in B-ALL. This results in deregulation of Ca^2+^ channels which have been shown to provide cell proliferation and apoptosis dysfunction [38,95]. TRPML2 channels were investigated in ALL patients since growing lines of evidence are focusing on the oncogenic role in cancer [96]. In B-ALL patients, the *TRPML2* channel gene is hypermethylated, and this aspect could be strongly implicated in ALL development; unfortunately, its role in ALL is still unclear [39].

### 3.2. TRP Channels in CLLs

CLL is the most common haematological cancer found in adults, with a higher incidence above 65 years old. It is a lymphoproliferative disorder characterised by a monoclonal mature expansion of CD5^+^CD23^+^ B cells in the peripheral blood, secondary lymphoid tissue, and bone marrow [97,98]. 

CD^5+^ cells could belong to the IgM-secreting B1 lineage of B cells, which are part of the innate immunity [89], but their origin is still debated. CLL patients are classified on the basis of genetic mutations of immunoglobulin heavy chain variable region (*IGHV*) genes, which encode for a B cell receptor (BCR) portion. IGHV-mutated cells are derived from antigen-experienced B cells which have transited through the germinal centre of secondary lymphoid organs, whereas the IGHV-unmutated cells appear to originate from naïve B-cells [89,99]. Previous data have demonstrated that B-CLL cells have an altered Ca^2+^ signalling pathway, which is involved in BCR engagement, survival, proliferation, differentiation, migration, antibody production, and, at high concentration, apoptosis [100,101]. Since Ca^2+^ is crucial for B-cell functions, the expression and functions of TRP channels have been investigated and are listed in Table 2. 

TRPV2 has been investigated in Jok-1 CLL cells and in its relative CD5-transfected Jok-E1A and Jok-E1B cells, with the CD5 protein transfected in the membrane or in the cytoplasm, respectively. CD5 is involved in B-CLL development through IL-10 production, and CD5^+^ B cells are the main source of this cytokine [102,103]. TRPV2 is expressed in all three cell models, with higher expression in Jok-E1A and Jok-E1B cells with respect to the Jok-1 cell line. Unfortunately, TRPV2 expression is not detectable in B-CLL or healthy patients [50].

TRPC1 overexpression was previously detected in CD5^+^ human B-lymphoblast cell lines [104], and it was linked to the increased Ca^2+^ entry and NFAT2 activation which leads to cytokine/chemokine production [105]. TRPC1 is expressed in the Jok-1 cell line and is upregulated in CD5-transfected Jok-E1A and Jok-E1B B-CLL cells. In B-CLL patients, the expression of TRPC1 was higher in pERK1/2^+^ patients, compared to pERK1/2^−^, with respect to the control samples (B and T cells from a healthy donor), with levels similar to the Jok-E1A cell line. TRPC1 is also involved in CD5-mediated IL-10 release. Indeed, through CD5 and TRPC1 silencing in pERK1/2^+^ B-CLL cells, IL-10 production is decreased, demonstrating that TRPC1 is involved in the production of anti-inflammatory cytokines, together with CD5 expression in a BCR-independent Ca^2+^-dependent way, promoting cell survival [50].

## 4. Hodgkin and Non-Hodgkin Lymphomas

Lymphomas are a heterogeneous group of lymphoid malignity characterised by different clinical behaviours and treatment response histories. Lymphomas are classified, according to WHO, in lymphoid neoplasms, originated from lymphoid precursor cells, and mature lymphoid cells; thus, they are divided into neoplasms of B-cell or T-cell origin. Mature lymphoid neoplasms include non-Hodgkin lymphomas (NHLs), whereas the Hodgkin lymphomas are grouped separately. Mature histiocytic and dendritic cell (HDC) tumours, whose origin is not related to lymphoid cells, are often considered with mature lymphoid neoplasms, given that they are involved in the lymphoid tissue [106,107,108]. 

### TRP Channels in HLs

Few data on the expression and functions of TRP ion channels in human lymphomas have been provided so far. However, some preliminary findings suggest that dysregulation of some members may be involved in lymphoma. In this regard, TRPM4 overexpression has been found in B-cell large lymphoma (DLBCL) [109]. 

Moreover, lovastatin, a statin that is able to reduce cancer incidence and mortality [110,111,112,113], has been found to inhibit human Daudi B cell lymphoma proliferation by reducing membrane cholesterol, intracellular ROS, and TRPC6 expression and activity, suggesting a contribution of this Ca^2+^ -permeable channel in lymphoma proliferation [53].

Finally, although in a non-human model, in canine B-cell lymphoma, a tumour clinically and histologically similar to human B-cell non-Hodgkin lymphoma, a predisposing locus on chromosome 5 associated with a 20% risk of developing B-cell lymphoma was identified by genome-wide association studies. Gene expression analysis of B cell lymphomas revealed that the *TRPC6* gene expression is almost completely reduced, together with other genes located in the same region [114].

## 5. TRPs as Promising Diagnostic, Prognostic, and Therapeutic Markers in the Clinical Management of Haematological Malignancies

It is now well accepted that alterations of TRP expression and functions are responsible for the impairment of cellular signalling pathways involved in cancer growth, metastasis, and chemoresistance [115]. In particular, dysregulations of TRPC, TRPM, and TRPV members have been mainly correlated with malignant growth and progression. For this reason, in recent years, many efforts have been spent to improve the knowledge about these channels and the ability to target them. Thus, they are considered promising tools to inhibit cancer progression and to ameliorate the diagnosis and overcome chemoresistance in cancer [29,116,117,118,119]. 

Interesting findings indicate and suggest that TRP channels could also be useful as diagnostic, prognostic, and therapeutic markers in the clinical management of haematological malignancies (Table 3).

In this regard, integrated methylome, transcriptome, and epigenetic analysis showed that the expression level of many genes is dysregulated in paediatric leukaemia, and among them, the presence of TRPC1, TRPC4, TRPC3, TRPM2, TRPM4, and TRPM8 also stands out. These TRP channels are repressed, and this indicates a reduced potential for cell–microenvironment interactions and apoptotic potential of the ALL cells [38,39]. In accordance with these data, the potential utility to add TRP as diagnostic tools is also confirmed by other studies. It has been found that TRPM4 is significantly overexpressed in diffuse large B-cell lymphoma (DLBCL), compared to normal germinal centre (GC) B cells and, in addition, it is more expressed in activated B-cell-like than in GC DLBCL [124]. Moreover, Hirai et al. demonstrated that TRPM8 positive neoplastic cells are mostly present in post-GC neoplasms but not in pre-GC or in the majority of GC neoplasms, suggesting TRPM8 as a marker to discriminate and diagnose reactive plasmablasts and mature B-neoplasms [123].

Finally, TRPM2 was found to be strongly upregulated in AML samples from patients with normal karyotypes or all AML mutational subgroups with respect to normal hematopoietic stem cells or common myeloid progenitor, suggesting the possibility to differentiate normal from neoplastic cells by using TRPM2 expression levels [49].

The potential prognostic impact of TRP channels in haematological malignancies has been recently highlighted. In DLBCL, the negativity of TRPM4 expression significantly correlated with better overall survival (OS) and progressive-free survival (PFS), compared with TRPM4 strong intensity. TRPM4 positivity was also associated with higher lactate dehydrogenase levels, higher Eastern Cooperative Oncology Group (ECOG) score, and stage III-IV. In addition, TRPM4-positive DLBCL patients treated with R-CHOP (cyclophosphamide, doxorubicin, vincristine, and prednisone) protocol displayed worse survival, consistent with its expression [124]. Moreover, a close relationship between TRPM8 and International Prognostic Index (IPI) scores was found in DLBCL patients, indicating that lower TRPM8 expression levels are associated with higher IPI scores [123].

In vivo studies underline the importance of developing new pharmacological approaches based on the targeting of TRP channels. In fact, mice injected with TRPM2-depleted leukaemia cells showed significantly reduced leukaemia, compared to controls, suggesting that these channels play an important role in leukemogenesis [49]. The targeting of TRP channels has been shown to affect leukaemia cells and the tumour microenvironment. It has been recently demonstrated that TRPV4, by acting as a volume receptor, is involved in bone marrow adipocyte remodelling in AML mice, and it is clear that the inhibition of this remodelling increases the survival in the AML mouse model [121].

Moreover, many in vitro studies, performed in patient-derived cells, demonstrated the ability of TRP-targeting therapy to inhibit cell proliferation and improve the effects of traditional chemotherapy in haematological malignancies. For instance, the TRPC3 channel blocker, Pyr3, enhances apoptosis induced by dexamethasone in ALL cells isolated from patients by altering calcium signalling, mitochondrial membrane potential, and ROS production [44]. In addition, the activation of TRPV1, by using the specific agonist RTX, reduced cell proliferation, blocked cell cycle, and increased apoptosis in T cells from ALL patients [37]. 

Obviously, further studies are necessary in order to develop and use drugs capable of modulating the TRP functions. However, interesting assumptions are gradually emerging. In fact, by molecular imaging methods, the in vivo potential of soricidin-derived peptides in targeting TRPV6-rich tumours has been evaluated [125]. In addition, an apoptosis-inducing TRPV1 nanoagonist containing semiconducting polymer nanoparticles (SPNs) as nanocarriers and CPS as the agonist has been developed to target TRPV1-positive cancer cells [43]. Finally, given that ongoing clinical trials specifically targeting TRPM4 have been approved in patients with stroke [126], it can be expected that, soon, other TRP-based therapeutic strategies may have an adequate safety profile and be applied in the field of cancer, including haematological malignancies.

## 6. Conclusions

Although TRP channels are found to be important in the carcinogenesis of many tumours so far, little is known about their involvement in leukaemia and lymphoma [34,117,119]. Findings gathered thus far demonstrate that TRPs display an oncogenic activity in haematological malignancies associated with alteration of their molecular expression profile. Given that targeting of TRPs could alter the signalling pathways associated with leukaemia, the evaluation of TRPs as promising biomarkers seems to be of particular interest in the diagnosis, prognosis, and/or treatment of haematological malignancies. 

Taken together, these data suggest that attention should be focused on these channels, and increasing efforts should be spent on better characterising their role and function in blood disorders to provide further oncologic targets for upgrading precision medicine.

## Figures and Tables

**Table 1:** Environmental activating stimuli and calcium permeability characteristics of TRP channels.

TRP	Ca^2+^ Selectivity	Activation Temperature	Exogenous Activators	References
TRPA1	Medium/Low	<17 °C	Caffeine, Cinnamaldehyde, Nicotine	[8,9,10]
TRPC1	Medium		Lanthanide ions (La^3+^, Gd^3+^), carbachol	[11]
TRPC3	Medium		OAG	[12]
TRPC6	Medium		OAG, Hyperforin, 2,4-Diacylphloroglucinol	[12,13,14]
TRPM2	Medium/Low	>38 °C	N-(p-amylcinnamoyl)anthranilic acid, Clotrimazole, Flufenamic acid	[15,16,17,18]
TRPM4	Non selective	15–35 °C	BTP2	[15,19,20]
TRPM7	Medium/Low		Naltriben	[21]
TRPM8	Medium	17–25 °C	Menthol, eucalyptol, geraniol	[6]
TRPML2	Non selective		SID24801657, SID24787221, ML2SA1	[22,23]
TRPV1	Medium/High	>42 °C	CPS, Piperine, Gingerol	[6,24]
TRPV2	Medium	(≈52 °C)	2-aminoethoxydiphenyl borate, diphenylyboronic anhydride, and *Cannabis sativa* derivatives	[6]
TRPV4	Medium	24–27 °C	Bisandrographolide A, Apigenin, 4-alpha-phorbol12,13-didecanoate	[6]
TRPV5	High		-	
TRPV6	High		CPS	[25]

Abbreviations: TRPA, transient receptor potential ankyrin; TRPC, transient receptor potential canonical; TRPM, transient receptor potential melastatin; TRPML, transient receptor potential mucolipidin; TRPV, transient receptor potential vanilloid; CPS, capsaicin; La^3+^, lanthanum; Gd^3+^, gadolinium; BTP2, N-[4-[3,5-Bis(trifluoromethyl)pyrazol−1-yl]phenyl]−4-methylthiadiazole−5-carboxamide; OAG, oleyl acetyl glycerol; - no data reported. Ca^2+^ selectivity scores of TRP channels, in accordance with PCa/PNa ratios, are considered as follows: high selectivity (10−100 score), medium selectivity (1−10 score), and low selectivity (<1 score). The data for these scores are found in the literature [26,27].

**Table 2:** Expression of TRP channels in leukaemia and lymphoma.

Cancer Type	Cell Line/Model	TRP	Methods	Functions	References
ALL	Jurkat	TRPV5/V6	RT-PCR, WB	Early endosome formation	[35,36]
	T-ALL patient	TRPV1	RT-PCR	Proliferation Cell death	[37]
	B-ALL patient	TRPC	FISH, RT-PCR	(?)	[38]
	B-ALL patient	TRPML2	RT-PCR	(?)	[39]
	Jurkat	TRPV2	PCR	Deformation stretch-activated current	[40,41]
	Jurkat	TRPM2	qRT-PCR, WB	ROS production	[42]
	Jurkat	TRPV1	RT-PCR	Cell proliferation	[37]
	Jurkat	TRPV6	RT-PCR, WB	Cell migration	[36,43]
	Nalm-6 Reh	TRPC3	Functional studies	ROS production, Cell death	[44]
AML	U937 THP1	TRPV2	RT-PCR, WB RT-PCR, WB	Cell growth, apoptosis, and cell cycle (?)	[45]
	THP1	TRPA1	Immunofluorescence	Macrophage cytotoxicity	[46,47]
	U937	TRPM2	RT-PCR, WB	Autophagy	[48,49]
CLL	Jok-1/E1A-E1B	TRPC1	Flow cytometry	Cytokine production	[50]
CML	32d and 32d-p210	TRPC1	WB	Cell proliferation	[51]
	K562	TRPM7	RT-PCR, IF	Erythroid differentiation	[52]
	K562	TRPV2	RT-PCR, WB	Cell death	[45]
Lymphoma	Daudi B-cell	TRPC6	WB	Cell proliferation	[53]

Abbreviations: ALL, acute lymphoblastic leukaemia; AML, acute myeloid leukaemia; CLL, chronic lymphocytic leukaemia; CML chronic myeloid leukaemia; TRPA, transient receptor potential ankyrin; TRPC, transient receptor potential canonical; TRPM, transient receptor potential melastatin; TRPML, transient receptor potential mucolipidin; TRPV, transient receptor potential vanilloid; ROS, reactive oxygen species; IF, immunofluorescence; FISH, fluorescence in situ hybridisation; RT-PCR, real-time polymerase chain reaction; WB, Western blot; (?) no conclusive data.

**Table 3:** Expression and functions of TRP channels in leukaemia and lymphoma patients.

Cancer Type	TRP	Effects	References
ALL	TRPC3	↓Glucocorticoid resistance	[44]
AML	TRPM2	↓OS ↓Doxo-sensitivity	[120]
	TRPV4	↑Chemotherapy efficacy	[121]
CLL	TRPC1	OS	[122]
Lymphoma	TRPM8	Prognostic	[123]
	TRPM4	↓OS ↓PFS	[124]

Abbreviations: ALL, acute lymphoblastic leukaemia; CLL, chronic lymphocytic leukaemia; TRPC, transient receptor potential canonical; TRPM, transient receptor potential melastatin; TRPV, transient receptor potential vanilloid; OS, overall survival; PFS, progression-free survival; ↑, increase; ↓, decrease.

## Data Availability

No new data were created or analyzed in this study. Data sharing is not applicable to this article.
